# Importance of assessing CK19 immunostaining in core biopsies in patients subjected to sentinel node study by OSNA

**DOI:** 10.1007/s00428-012-1241-z

**Published:** 2012-05-04

**Authors:** Felip Vilardell, Anna Novell, Javier Martin, Maria Santacana, Ana Velasco, M. J. Díez-Castro, Dolors Cuevas, M. Jose Panadés, Serafin González, Antonio Llombart, Edelmiro Iglesias, Xavier Matias-Guiu

**Affiliations:** 1 Department of Pathology and Molecular Genetics, Hospital Universitari Arnau de Vilanova, Lleida, Spain; 2 Department of Nuclear Medicine, Hospital Universitari Arnau de Vilanova, Lleida, Spain; 3 Department of Medical Oncology, Hospital Universitari Arnau de Vilanova, Lleida, Spain; 4 Unit of Breast Pathology, Department of Surgery, Hospital Universitari Arnau de Vilanova, Lleida, Spain; 5 Servei d’Anatomia Patològica i Institut de Recerca Biomèdica de Lleida, Hospital Universitari Arnau de Vilanova, Alcalde Rovira Roure 80, 25198 Lleida, Spain

**Keywords:** Breast carcinoma, OSNA, CK19, Luminal, “Basal-like”

## Abstract

Analysis of sentinel lymph node (SLN) by means of One-Step Nucleic Acid Amplification (OSNA) is being used increasingly as a very sensitive and quick method for intraoperative axillary staging in patients with breast cancer. This molecular diagnostic assay detects the expression level of cytokeratin 19 (CK19), a luminal epithelial cell marker broadly expressed in most breast carcinomas and not normally found in lymph nodes. Almost all breast cancers express this cytoskeleton protein, but some breast tumors have been found to lose the expression of CK19. CK19 immunostaining in core biopsies has been recommended in selecting patients eligible for OSNA analysis because SLNs with metastatic involvement by CK19-negative breast cancers may result in a false negative result by OSNA. However, the real frequency of CK19-negative breast cancer has to be elucidated. In this study, we have assessed the frequency and molecular profile of CK19-negative breast carcinomas in three series of cases. The first is a prospective series of 197 breast carcinomas, 111 of which were subjected to SLN evaluation by OSNA. The second is a retrospective series of 41 triple-negative (TN) breast carcinomas, and the third is a retrospective series of 68 breast cancer patients (matched core biopsies and metastatic lymph nodes) that had been evaluated by conventional procedures before the OSNA methodology was adopted in our institution. Our results not only demonstrate that lack of expression of CK19 is infrequent in breast cancers but also that performing CK19 immunohistochemical staining is important on diagnostic core biopsies in taking the decision of using OSNA methodology in the evaluation of sentinel nodes in breast cancer patients.

## Introduction

In the last 10 years, breast carcinoma has been categorized by means of cDNA microarray studies [1, 2] in five subtypes: luminal A and B, Her2, basal-like, and the more controversial normal breastlike. Luminal breast cancers show high expression levels of several luminal epithelial cell markers like CK7, CK8, CK18 and CK19, MUC1, bcl-2, estrogen receptors (ER), progesterone receptors (PR), GATA3, epithelial cell adhesion molecules, and lower expression levels of basal CKs (CK5, CK14, and CK17). In contrast, breast cancers with a basal-like phenotype [3] are characterized by a protein expression pattern which is similar to that of myoepithelial cells, including a higher expression of high molecular weight cytokeratins, such as CK5, CK14 and CK17, smooth muscle markers, p63, CD10, EGFR/HER1, or beta-4 integrin, among others, and a lower expression of ER, PR, or HER2.

Currently, in clinical practice, the cytokeratin expression pattern of breast tumors usually is not assessed, and breast cancers are, as a rule, immunohistochemically classified into four immunophenotypes with very important implications [4, 5] in order to decide the more appropriate therapeutic approach: luminal A (ER + and/or PR+, HER2−), luminal B (ER + and/or PR+, and Ki67 > 14 % or HER2+), HER2 overexpressing (ER−, PR−, HER2+), and triple-negative (TN) phenotype (ER−, PR−, HER2−) [6]. Most TN breast cancers fit within the basal-like subgroup, although these two terms are not synonymous, and immunohistochemical assessment of EGFR and basal cytokeratins 5 and 6 is usually needed for better profiling of the basal-like phenotype among the TN tumors [7].

Sentinel lymph node (SLN) analysis by means of the One-Step Nucleic Acid Amplification (OSNA) assay is an increasingly used procedure for intraoperative staging of breast cancer patients [8]. The OSNA assay [9] (Sysmex, Kobe, Japan) is based on real-time amplification and quantitation of CK19 mRNA in homogenized samples of lymph nodes. In spite of the differences in cytokeratin expression pattern between luminal and basal-like breast cancers, luminal CK19 was chosen as the most suitable marker for identifying breast cancer metastases in lymph nodes by means of OSNA because virtually all (98.2 %) primary breast cancers were found to express this cytoskeletal protein [10]. CK19 mRNA has been described as having the highest sensitivity at nearly 90 % and was chosen as the best marker for OSNA assay among 45 potential mRNAs [8]. However, CK19 is not expressed in a small proportion of breast carcinomas including tumors with a luminal phenotype. Abd El-Rehim reported in 2004 [11] a consecutive series of 1944 breast carcinomas with 3.4 % (42/1230) of ER-positive tumors lacking CK19 expression. Similarly, Parikh et al. [12] reported in a cohort of 158 young women with stage I/II breast cancer, absence of CK19 expression in 6 % (4/66), and 30 % (21/62) of carcinomas with non-TN and TN phenotype, respectively.

In our institution, OSNA procedure is complemented with touch imprints as a quality control procedure. Between February 2009 and April 2011, nearly 450 breast carcinomas have been subjected simultaneously to OSNA and touch imprints. Concordant results were obtained in all but 13 of the cases, including three cases with isolated cells, eight cases with micrometastases, and one case with macrometastases, all of them regarded as negative by means of the touch imprints. One more discrepant case resulted in a SLN with malignant cells observed in the touch imprints but not detected by OSNA. This case turned out to be a grade I CK19-negative breast carcinoma with a luminal A immunophenotype and showed a high expression level of both ER and PR and a low Ki-67 proliferative index (4 %). The Her-2 expression was scored as negative (1+). A lymph node metastasis was seen in a touch imprint from the SLN but had not been detected by molecular procedures. The absence of expression of CK19 was confirmed by Sysmex Europe GmbH. The diagnostic core biopsy obtained several weeks before SLN analysis was also CK19-negative. Since this case was found, we routinely checked the status of CK19 in all newly diagnosed breast cancers on the diagnostic core biopsy. When CK19 expression is confirmed in the core biopsy, analysis of SLN is made by OSNA analysis in parallel with a touch imprint. If CK19 is negative, analysis of SLN is done by frozen section, touch imprints, and evaluation of formalin-fixed and paraffin-embedded samples, stained with hematoxylin and eosin (H&E), and immunostained with the pan-cytokeratin antibody AE1/AE3.

In order to assess the practical consequences of the absence of CK19 expression in breast cancer and to validate our strategy, we have conducted a prospective and retrospective study in order to determine the frequency of CK19-negative tumors within each of the four distinct immunophenotypes of breast carcinoma. Our results support the notion that the absence of expression of CK19 is rare in breast cancer but does occur in carcinomas with either a luminal or basal-like phenotype. Therefore, evaluation of CK19 in the diagnostic core biopsy may help in taking the decision as to the use of OSNA methodology in the evaluation of sentinel nodes in breast cancer patients.

## Materials and methods

### Patients and tumor specimens

Three different series of cases were studied. The first is a prospective consecutive series of 197 breast cancer core biopsies of patients who were diagnosed in Hospital Universitari Arnau de Vilanova, Lleida, Spain between April 2010 and April 2011. The mean age was 62.0 years (range 30 to 96 years). The histology of the cases included 182 (92.4 %) ductal breast carcinomas NOS, six (3.0 %) lobular breast carcinomas, and nine (4.6 %) belonging to other histologic subtypes. Tumor mean size was 18.2 mm. From them, 111 patients were subjected to sentinel node biopsy and OSNA evaluation.

Secondly, a retrospective series that included 41 TN breast carcinomas surgically resected from 2000 through 2010 was evaluated for CK5/6 and EGFR to identify breast cancers that have a basal-like phenotype. Subsequently, the CK19 status was checked in all the tumors from this series.

Thirdly, we included a retrospective series of 68 patients in which SLN was found to be positive for metastasis by conventional pathological examination (touch imprints, frozen sections, and H&E and cytokeratin expression in formalin-fixed paraffin-embedded material) before the OSNA procedure was implemented in our institution on February 2009. Material available for these cases included sentinel node specimen and matched primary tumors.

### OSNA

The OSNA assay for CK19 mRNA was introduced in our pathology department on February of 2009 as a routine method for analysis of SLN of breast cancer patients after a multicentric validation study performed under the auspices of Sociedad Española de Senología [12]. Between April 2010 and April 2011, 111 breast cancer patients from the first series were subjected to OSNA analysis of sentinel node. The OSNA protocol consisted of homogenization of tissue in a mRNA-stabilizing solution (Lynorhag, pH 3.5 Sysmex®) and subsequent isothermal (65 °C) amplification of cytokeratin 19 (CK19) using the Lynoamp amplification kit (Sysmex®) through a reverse transcriptase amplification assay (RT-LAMP) in a gene amplification detector RD-100i (Sysmex®) in compliance with the protocol previously described [8, 9]. The technique uses six primers, which increase the specificity and the speed of the reaction. In the OSNA assay, cases showing mRNA CK19 levels greater than 250 copies/μL were considered positive and were classified as micrometastases (number of copies >250 copies/μL <5,000 copies/μL) or macrometastases (number of copies >5,000 copies/μL) following system specifications based on prior calculations. Cases identified as “negative” (<250 copies/μL) by the system were further classified as isolated tumor cells (ITC) (no. of copies per microliter >100 but less than 250) or true negative if the number of copies per microliter was <100.

A positive OSNA reaction was found in 54 of the 111 cases of the first series (33 macrometastases, 19 micrometastases, and two ITC). In all cases, the OSNA analysis has been complemented with touch imprints, which are performed simultaneously with OSNA procedure. Concordance between OSNA results and touch imprints was observed in all but one of the patients, the latter corresponding to the first CK19-negative breast cancer mentioned above.

### Immunohistochemistry

#### ER, PR, Ki-67, and Her-2

The immunophenotype of the three series of tumors was assessed in real time by means of immunolabeling for ER, PR, Ki-67, and Her-2 on the diagnostic core biopsies. For immunohistochemical analysis of ER, PR, and Ki-67 in the prospective consecutive series, the tissue sections were deparaffinized and endogenous peroxidase activity was blocked with Peroxidase-Blocking Reagent for 10 min. For ER and PR immunostaining, epitope retrieval was performed by heating at 95 °C in Target Retrieval Solution, High pH (50× Tris/EDTA buffer, pH 9) of Dako® for 20 min. The slides were subsequently incubated for 25 min with monoclonal mouse antihuman ERα antibody (clone 1D5, isotype IgG1, kappa) for ER staining and with monoclonal mouse antihuman PR antibody (clone PgR 636, isotype IgG1, kappa) for assessment of PR expression. The expression of ER and PR was graded semiquantitatively by considering the percentage and intensity of the staining applying the following formula: Histoscore = 1 × (% light staining) + 2 × (% moderate staining) + 3 × (% strong staining). The Allred score 7 is used as well. In order to assess the proliferative index, antigen retrieval was performed by heating at 95 °C in Target Retrieval Solution, High pH (50× Tris/EDTA buffer, pH 6.1) of Dako® for 20 min and incubation with monoclonal mouse antihuman Ki-67 antigen (clone MIB-1) for 20 min. Ki-67-positive nuclei were scored in the tumor areas showing the highest number of stained nuclei. In all cases, the slides were incubated with EnVision™ FLEX of Dako® as secondary antibody for 20 min, with chromogen diaminobenzidine tetrahydrochloride for 10 min and finally, counterstained with hematoxylin, dehydrated with ethanol, and mounted. Her-2 immunolabeling was performed following the HercepTest™ of Dako®. The antigen retrieval was by heating at 97 °C for 40 min with HercepTest™ Epitope Retrieval Solution (containing detergent 10×) (0.1 mol/L citrate buffer with a detergent). The slides were incubated with the primary antibody HercepTest™ Rabbit Anti-Human HER2 Protein for 30 min and with HercepTest™ Visualization Reagent for 30 min as secondary antibody.

#### Basal-like immunophenotype

All tumors which showed a TN phenotype were subsequently immunostained for EGFR and CK5/6 in order to characterize them as “basal-like” or “non basal-like.” EGFR immunostaining was performed using the kit EGFR pharmDx of Dako®. The samples were digested for 5 min with proteinase K for antigen retrieval and incubated for 30 min with the primary antibody Mouse Antihuman EGFR protein. Subsequently, they were incubated for 30 min with the secondary antibody Labelled Polymer-HRP. CK5/6 was assessed by means of the PT Link Pre-treatment Module of Dako®, using Target Retrieval Solution, High pH, for 20 min for antigen retrieval and a 20-min incubation with the primary antibody Monoclonal Mouse Anti-Human clone D5/16 B4. The slides were incubated with EnVision™ FLEX of Dako® as secondary antibody for 20 min. CK 5/6 and EGFR were scored following qualitative criteria. TN tumors showing either CK5/6 or EGFR expression, even if focal, were regarded as tumors with a “basal-like” phenotype [7].

#### CK19 immunostaining

Finally, CK19 was assessed by incubation with the RCK108 primary antibody for 20 min. Methodology was similar to that performed for ER and PR, and the samples were incubated with EnVision™ FLEX of Dako® as secondary antibody for 20 min.

#### Statistics

For statistical evaluation of the data reported in Table 2, the Statistical Package for Social Science (SPSS) software was used. The contingency tables for clinicopathological correlations were analyzed by means the *χ* ^2^ test.

## Results

Results are summarized in Tables 1 and 2.

**Table 1:** Characteristics of the prospective series of 197 breast cancer cases in which CK19 expression has been assessed

Age (years)	
Mean	62.05
Median ± SD	63 ± 15.25
Range	30–96
Histology	
Ductal	182 (92.4 %)
Lobular	6 (3.0 %)
Other	9 (4.6 %)
Tumor size (mm)	
Mean	18.2
T1 (<2.0)	110
≥T2 (≥2.0)	54
Tumor grade	
Grade I	56 (28.4 %)
Grade II	104 (52.7 %)
Grade III	15 (7.6 %)
NA	22
Nodal status	
Total assessed	174 (87.9 %)
Negative	77 (38.9 %)
Positive	97 (49.0 %)
ER status	
Negative	37 (18.8 %)
Positive	160 (81.2 %)
Her2 status	
Negative	170 (86.3 %)
Positive	27 (13.7 %)
Ki67	
Low (<14 %)	61 (31.0 %)
High (>14 %)	136 (69.0 %)
Immunophenotype	
Luminal	
—Luminal A	57 (28.9 %)
—Luminal B (ER + and Her2+ or Ki67 > 14 %)	103 (52.3 %)
—Total luminal	160 (81.2 %)
Her-2	14 (7.1 %)
Triple-negative	23 (11.7 %)
Cytokeratin 19 status	
Positive	191 (96.5 %)
Negative	6 (3.0 %)
Undone	5 (0.5 %)

**Table 2:** Association between CK19 status and patient features

	CK19 status		
	Positive	Negative	*p* value
Age (years)			
<Mean	89	2	0.048
>Mean	102	4	
Histology			
Ductal (NOS)	176	6	0.920
Lobular	6	0	
Other	9	0	
Tumor size (cm)			
T1 (<2.0)	105	5	0.249
T2 (≥2.0)	54	0	
Tumor grade			
Grade I	54	2	0.123
Grade II	101	3	
Grade III	15	1	
Nodal status			
Negative	77	0	0.096
Positive	92	5	
ER status			
Negative	36	1	0.000
Positive	155	5	
Her2 status			
Negative	161	6	0.036
Positive	27	0	
Triple-negative status			
Positive	22	1	0.791
Negative	169	5	
Immunophenotype			
Luminal A (ER+, Ki67 < 14 %, Her-2−)	55	2	0.754
Luminal B (ER+, Ki67 > 14 % or Her-2+)	100	3	
Her-2	14	0	
Triple-Negative	22	1	
Immunophenotype			
Luminal A and B	155	5	0.000
Her-2	14	0	
Triple-negative	22	1	

### Prospective series of 197 breast carcinomas

First, we evaluated a prospective series of 202 consecutive, unselected breast cancers, 111 of which were subjected to SLN evaluation by OSNA. In that series, the expression of CK19 had been assessed in 197 cases. This series comprised 57 (28.9 %) luminal A cases (ER + and/or PR+, Ki67 < 14 %, Her2−), 103 (52.3 %) luminal B cases (ER + and/or PR+, Ki67 > 14 % or Her2+), 14 (7.1 %) Her2 cases (ER−, PR−, Her-2+), and 23 (11.7 %) TN cases, with 16 (7.9 %) basal-like tumors by means of immunolabeling with CK5/6 and EGFR.

**Fig. 1:**
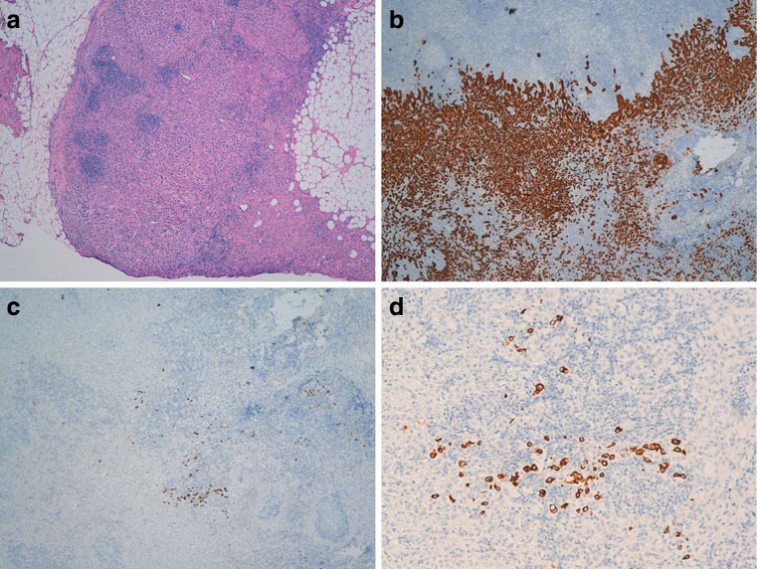
Sentinel lymph node from the fourth luminal A CK19-negative breast carcinoma. **a** Lymph node section showing a macrometastasis of 9 mm in maxim diameter. **b** View of the metastatic population of cells by means of CKAE1/AE3 pan-cytokeratin immunostaining of the same section. CK19 immunostaining of the same lymph node section (**c** and **d**). Only a small proportion of metastatic cells express CK19. **a** H&E staining, original magnification × 40. **b** Original magnification × 200. **c** Original magnification × 40. **d** Original magnification × 200

In this series, a total of six (3.04 %) CK19-negative breast carcinomas were found. Five of the six carcinomas lacking CK19 expression were positive for ER and regarded as carcinomas with a luminal phenotype (5/160, 3.1 %), two of them with a luminal A phenotype and the remaining three with a luminal B phenotype. The sixth CK19-negative case was a TN carcinoma, with a basal-like phenotype (1/24, 4.2 %). The first luminal-type carcinoma was the case described in the “Introduction,” a grade I breast carcinoma with high expression of ER and PR, low Ki67 index (luminal A), and a metastatic sentinel node that was misinterpreted by OSNA. The second CK19-negative case, with a luminal A phenotype, was not treated in our center. In the third luminal-type tumor (luminal B), lymph node metastases were demonstrated by means an axillary fine-needle aspiration as well as in the basal-like one. In the fourth and fifth luminal carcinomas (luminal B) lacking expression of CK19, the SLNs were examined by means of frozen and paraffin-embedded sections. Such a decision was taken after CK19 staining turned out negative in the diagnostic core biopsy. A micrometastasis was detected in one case by means of immunolabeling for cytokeratins using the AE1/AE3 antibody and a macrometastasis (9 mm) in the other case, which was seen in the frozen section. Then, we checked the expression of CK19 in these two metastases. The micrometastasis from the fourth CK19-negative luminal tumor was CK19-negative as well. The macrometastasis from the fifth CK19-negative luminal carcinoma, however, showed some isolated CK19-positive cells (Fig. 1). When the immunohistochemical profile of the tumors had been initially assessed in core biopsies samples, we compared this with the CK19 status of this primary tumor on the surgical specimen to rule out the possibility that the core biopsy was not representative for the whole tumor in terms of CK19 expression. This primary carcinoma was mostly CK19-negative, but there were some small areas with immunolabeling for CK19, and these areas were not present in the core biopsy.

### Retrospective series of 41 triple-negative (TN) breast carcinomas

The series of 41 TN breast carcinomas consisted of 30 tumors with a basal-like phenotype and nine carcinomas with a nonbasal-like TN phenotype. In two cases, we could not test retrospectively the expression of CK5/6 and EGFR in order to further characterize these as basal-like or “nonbasal-like. No tumor from this series was CK19-negative.

### Retrospective series of 68 cases with metastases in SLN before OSNA implementation

SLN was evaluated in 211 breast cancer cases before the OSNA methodology was adopted in our institution. All the SLNs had been studied by means conventional pathological and immunohistochemical examination, and the cases were retrospectively reviewed in order to find CK19-negative metastases which could have originated false negative results by OSNA. Eighty-six SLNs had been positive for metastasis, 23 with micrometastases, and 63 with macrometastases, but in 18 cases, the metastatic focus was exhausted in the remaining material. Of the other 68 cases with metastatic foci still present, all but one case was CK19-positive. In this case, the expression of CK19 was checked in the surgical specimen of the matched primary tumor, and the tumor was homogeneously CK19-negative.

## Discussion

The OSNA methodology for detection of axillary metastasis in breast cancer is becoming a standard procedure and is based on measuring the expression level of CK19 in the SLN. CK19 was chosen as the optimal molecule because of its broad expression in most breast carcinomas, without notable expression in hematopoietic cells [10]. Validation studies published to date [13–17] show that OSNA is a reliable quantitative test of intraoperative metastasis detection in SLNs of breast cancer patients.

In general, breast cancer is regarded to arise from luminal epithelial cells of the terminal duct lobular unit (TDLU) which express several cytokeratins such as CK7, CK8, CK18, and CK19 [1]. This suggests that virtually all breast carcinomas express CK19, but still there is the possibility that some breast cancers loose CK19 expression during tumor development and progression. This may be a challenge for the accuracy of OSNA in evaluating SLN. Moreover, a smaller group of breast carcinomas express some cytokeratins normally found in the outer basal layer of myoepithelial cells of the mammary ductules, such as CK5, CK14, and CK17, and, for this reason, are designated basal-like [3, 6]. Consequently, breast cancers lacking CK19 expression might occur only in the basal-like subtype of breast cancer or might also contain luminal breast carcinomas with loss of CK19 expression.

A few studies have addressed the frequency of CK19-negative breast cancers. Parikh et al. (2008) [12] reported lack of CK19 expression in 20.5 % of 158 breast carcinomas in a tissue microarray. Moreover, they found a statistically significant association between lack of CK19 expression and the TN phenotype (30 % of TN breast cancers were CK19-negative). However, four of their 27 CK19-negative tumors were not triple-negative, which in their series amounted to 6 %. Similarly, Abd El-Rehim et al. (2004) [11] reported lack of CK19 expression in 3.4 % of luminal (ER+) breast cancers. Very recent works [18] have assessed the expression of CK19 in different histological types of breast cancer but without consideration of their molecular profile.

A case of discordance between metastatic cells seen in a touch imprint of a SNL but not detected by means of the OSNA assay, allowed us to find a grade I ductal carcinoma with a luminal A phenotype which did not express CK19. The lack of CK19 expression was confirmed by Sysmex Europe GmbH. From then on, we assessed the CK19 status of all newly diagnosed breast carcinomas. To date, from a consecutive series of 197 breast cancers in which CK19 expression was assessed, we found lack of CK19 expression in 3.5 % (2/57) of breast cancers with a luminal A phenotype (ER/PR+, Her2−, Ki67 < 14 %), 2.9 % (3/103) of breast cancers with a luminal B phenotype (ER/PR + and Ki67 > 14 % or Her2+), and 4.2 % (1/24) in triple-negative carcinomas. We did not find any CK19-negative tumor with a pure Her2-positive phenotype. We conclude from these results and literature review that absence of CK19 expression can be expected in around 4 % to 7 % of breast carcinomas with either a luminal or nontriple-negative phenotype (which can include the Her2 phenotype). It is conceivable that in some breast cancers, the lack of CK19 expression might be due to an absence of translation of the transcript CK19 mRNA. In these cases, the OSNA assay would be able to detect and amplify CK19 mRNA in the absence of protein. Nonetheless, it seems rational to check, as a rule, the expression of CK19 by immunohistochemistry in all newly diagnosed breast carcinomas in order to decide whether the analysis of SLN should preferably be done using the OSNA assay or conventional procedures. In one of the five luminal carcinomas lacking CK19 expression on the core biopsy, a macrometastasis was found in the SLN by means of cytology and frozen section examination. Just a few isolated cells of this macrometastasis expressed CK19. OSNA assay might have interpreted as isolated cells this macrometastasis. Interestingly, in this case, evaluation of CK19 in the surgical specimen also demonstrated the occasional presence of CK19-positive cells, which had not been seen in the diagnostic core biopsy, thus demonstrating that CK19 expression heterogeneity can also be an additional problem in the strategy of using CK19 evaluation in the core biopsy as an indicator of eligibility for OSNA evaluation in SLNs.

Furthermore, we decided to study retrospectively a series of 68 breast cancer patients that underwent SLN evaluation by conventional pathological assessment before OSNA assay was implemented in order to assess the number of cases that might have been misinterpreted as negative had they been assessed by OSNA because of lack of CK19 immunostaining. We found one case of SLN metastasis which was CK19-negative, which translates into a 1.4 % of false negative rate of SLN assessment using the OSNA assay. Interestingly, in this case, CK19 was not expressed in the core biopsy, suggesting that the absence of CK19 expression in the SLN might have been predicted by the evaluation of the core biopsy.

Based on our results, we conclude that even though the overall prevalence of CK19-negative breast carcinomas is very low, they do occur not only in tumors with a triple-negative or basal-like phenotype but also in breast cancers with a luminal phenotype, in which expression of luminal cytokeratins including CK19 is expected to be the rule. Therefore, expression of CK19 should be routinely assessed in all newly diagnosed primary breast carcinomas in order to decide whether the intraoperative axillary staging should be performed by means of cytology and pan-cytokeratins immunostaining on SLN sections or by means of real-time amplification and quantitation of CK19.
